# Long-term outcomes of patients with kidney and intestine-containing graft co-transplantation

**DOI:** 10.1016/j.intf.2024.100005

**Published:** 2024-07-09

**Authors:** Simran Shah, Julie Hong, Youjia Li, Keli Wang, Shaheed Merani, Joshua Weiner

**Affiliations:** a Columbia University Vagelos College of Physicians and Surgeons, New York, NY, United States; b Center of Translational Immunology, Department of Medicine, Columbia University, New York, NY, United States; c Columbia University School of Engineering and Applied Science, New York, NY, United States; d Department of Surgery, University of Nebraska, Omaha, NE, United States; e Department of Surgery, Columbia University Vagelos College of Physicians and Surgeons, New York, NY, United States

**Keywords:** Kidney transplantation, Intestine transplantation, Kidney failure, Co-transplantation

## Abstract

**Introduction:**

Transplant is the standard of care for patients with kidney or intestine failure. However, in patients with both kidney and intestine failure, it is unclear whether simultaneous or delayed transplant is preferred. This study explores survival and graft-failure outcomes for simultaneous (SK), delayed (DK), and no kidney (NK) transplantation in the setting of simultaneous intestinal and kidney failure.

**Methods:**

Scientific Registry of Transplant Recipients was queried between January 1994 and December 2023 for all first-time intestine transplant recipients undergoing preoperative peritoneal or hemodialysis. Patients were grouped by kidney transplant timing. Bivariate analyses were conducted on demographics, including age, gender, race, ethnicity, insurance status, and diabetes status. Censured survival curves were developed for overall survival and intestinal graft failure-free survival. All statistics were conducted on SAS Academic.

**Results:**

A total of 54 patients (8 NK, 39 SK, 7 DK) were included. DK patients had both greater overall survival (p = 0.029) and graft failure-free survival (p = 0.020). Adjusted models with survival time measured from time of kidney transplant showed a clinically significant trend for both graft and overall survival for DK patients.

**Conclusions:**

Patients with end stage renal disease and intestinal failure may benefit from delayed kidney transplantation. As this is a rare situation with few instances in large databases, further research would be beneficial.

## Introduction

Intestine transplantation (ITx) is the accepted therapy for patients with intestinal failure (IF) for whom parenteral nutrition is no longer a viable long-term option. Kidney transplantation (KTx) is the accepted therapy for patients with end-stage renal disease who do not have medical or surgical contraindications to transplantation. However, there is no current standard of care for patients with both end-stage IF and renal failure.

Simultaneous kidney transplantation is a common practice for other solid organ graft recipients with concomitant renal failure and has been extensively studied for heart [1], [2], [3], liver [4], [5], [6], lung [7], and, most commonly, pancreas [8] recipients. The strategy of delayed kidney transplantation that prioritizes patients who recently had an organ transplant has also been evaluated in liver [9] and heart transplantation [8]. However, while co-transplantation of kidney and intestine grafts is occasionally performed successfully for patients with end-stage renal disease (ESRD) who are undergoing ITx [10], [11], there is no large series analysis to indicate whether these patients benefit from simultaneous versus delayed transplantation of the kidney. To provide guidance for the management of this subset of patients, we set out to evaluate the outcome of patients’ renal allograft function during simultaneous and delayed kidney transplant in the setting of intestine transplantation.

## Material and methods

### Study design

This study used data from the Scientific Registry of Transplant Recipients (SRTR). The SRTR data system includes data on all donor, wait-listed candidates, and transplant recipients in the US, submitted by the members of the Organ Procurement and Transplantation Network (OPTN). The Health Resources and Services Administration (HRSA), U.S. Department of Health and Human Services provides oversight to the activities of the OPTN and SRTR contractors. The data reported here have been supplied by the Hennepin Healthcare Research Institute (HHRI) as the contractor for the Scientific Registry of Transplant Recipients (SRTR). The interpretation and reporting of these data are the responsibility of the author(s) and in no way should be seen as an official policy of or interpretation by the SRTR or the U.S. Government.

A retrospective clinical study using the SRTR data was designed to evaluate intestinal transplant recipients between January 1995 and December 2023. The SRTR datasheet for all intestinal transplant recipients (tx_in) served as the primary reference and was supplemented with data from both the SRTR datasheet for all kidney transplant recipients (tx_ki) as well as intestinal transplant recipients with long-term follow up (txf_in). Variable PERS_ID, which is a deidentified stand-in for a patient’s social security number, was used to confirm correct cross-reference. For patients with duplicate entries due to a repeat intestinal transplantation, the earliest encounter was included. Request to utilize this retrospective data for research was reviewed and approved by SRTR. All variables were referenced using SRTR Data Dictionary, which is available online.

### Patients and study groups

Patients with preoperative peritoneal dialysis or hemodialysis who were listed for kidney transplant prior to intestinal transplant were included. Dialysis status was determined using the CAN_DIAL variable which contained data for 2349 out of 3455 ITx patients (67.99 %). Patients with any kidney transplant as per datasheet tx_ki were grouped by kidney transplant timing in relation to the date of first intestinal transplant. Patients who underwent kidney transplant within seven days of intestinal transplant were categorized as simultaneous kidney transplantation (SK). Patients with kidney transplant following seven days were considered to have had a delayed kidney transplant (DK). Patients who received no kidney transplant (NK) were labeled as such.

### Statistical analysis

Bivariate analyses, which included ANOVA, Kruskal-Wallis, and Fisher Exact tests were conducted on demographics, including candidate age, gender, race, ethnicity, insurance status, diabetes status, Estimated Post Transplant Score (EPTS), and donor age. EPTS was approximated using the online calculator provided by OPTN [12]. Due to a majority of patients with missing variables in diabetes status (33 out of 54 in tx_in datasheet) and dialysis start date (27 out of 54 missing), all patients with missing information was assumed to have no diabetes, and all patients were assumed to have had dialysis start 0 years ago. There were not enough data points to only use patients who had dialysis start dates.

Patient survival and intestinal graft failure-free survival were compared between groups using censored survival curves. A graft failure event was inclusive of graft failure as well as death. Outcomes of interest, which were days to graft failure and days to recipient death, were limited to 2000 days. This was felt to be appropriate, as patient sample size grew exceedingly small after the five-year timepoint.

All statistical analyses were conducted on SAS Academic.

## Results

A total of 54 patients (8 NK, 39 SK, 7 DK) were included (Table 1). Time from listing to intestinal transplant had a median of 119 days [IQR 47–259, Range 1–1959]. In the DK group, time from intestinal transplant to kidney transplant had a median of 3087 days [IQR 1252–4562]. Post-kidney-transplant survival as estimated by EPTS score was comparable between patients of all three groups (p = 0.986).

**Table 1:** **Bivariate analysis of demographic variables for intestinal transplant recipients for three groups of kidney transplant timing** (No Kidney [NK], Simultaneous Kidney [SK], Delayed Kidney [DK]). Table 1A depicts continuous variables with minimal skew on univariate analysis (− 0.5 to 0.5), analyzed by ANOVA. Table 1B depicts Kruskal-Wallis analysis of continuous variables with skew on univariate analysis (< − 0.5 or > 0.5). Table 1C depicts Fisher Exact Test analysis of categorical variables.

**A. Continuous variables – ANOVA**						

	**Mean**	**SD**	**p-value**			

**Recipient Age**			0.872			
* No Kidney*	34.5	16.4				
* Simultaneous Kidney*	33.4	19.2				
* Delayed Kidney*	29.7	20.3				
**Recipient Albumin**			0.244			
* No Kidney*	3.0	0.7				
* Simultaneous Kidney*	3.1	0.7				
* Delayed Kidney*	3.6	0.8				
			

**B. Continuous variables – Kruskal Wallis**						

	**Median**	**Interquartile range**	**p-value**			

**Recipient Body Mass Index**		0.454				
* No Kidney*	24	19-30				
* Simultaneous Kidney*	22	20-26				
* Delayed Kidney*	20	18-23				
**EPTS**			0.986			
* No Kidney*	10	8-19				
* Simultaneous Kidney*	12	4-20				
* Delayed Kidney*	17	6-23				


**C. Categorical variables –Fisher Exact Test**						

	**No kidney**	**Simultaneous Kidney**	**Delayed Kidney**	**NK vs SK p-value**	**NK vs DK p-value**	**SK vs DK p-value**

**Gender**				0.706	0.619	1.000
* Female*	5	20	3			
* Male*	3	19	4			
**Race**				0.196	1.000	0.496
* Asian*	0	0	0			
* Black*	2	3	1			
* Native American*	0	0	0			
* Pacific Islander*	0	0	0			
* White*	6	36	6			
**Ethnicity**				0.436	1.000	0.398
* Hispanic*	1	2	1			
* Non-Hispanic*	7	37	6			
**Diabetes**				0.405	1.000	1.000
* Yes*	1	1	0			
* No*	3	13	3			

Detailed bivariate analyses are summarized in Table 1. No differences were identified between kidney transplant timing groups (NK, SK, DK) in recipient age (p = 0.872), body mass index (BMI) (p = 0.454), and albumin (p = 0.244). Furthermore, pairwise Fisher Exact Tests did not reveal differences by group in gender (p > 0.05), race (p > 0.05), ethnicity (p > 0.05), and diabetes (p > 0.05). Wait time for intestine transplant also did not significantly vary by group, suggesting no significant differences in graft donor pools (median [25–75th quartile]): DK (111 days [68.5, 185]), NK (110 days [41.5, 149]), SK (156 days [44.5, 269]) (Kruskal Wallis p = 0.505).

**Fig. 1:**
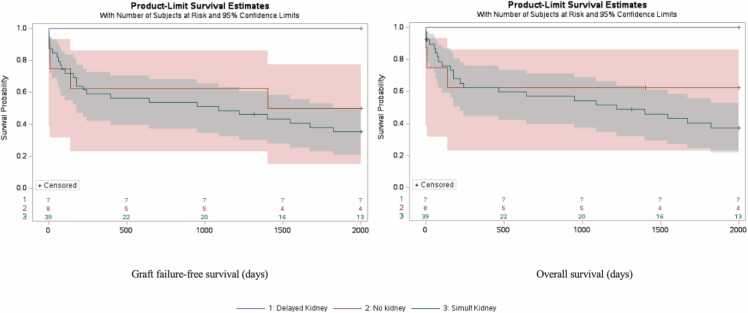
**A. Kaplan-Meier Survival Curve for graft failure-free survival within 2000 days**. Patients were grouped by timing of kidney transplant in relation to timing of intestinal transplant. Number at risk displayed by group in 500-day intervals. Censored data marked with a cross. Log-rank analysis of curve revealed significant differences between groups (p = 0.020). 1-, 3-, and 5-year survival ratios by group were highest for delayed kidney group (100 %, 100 %, 100 %), lower for no kidney (63 %, 63 %, 50 %), and lowest for simultaneous kidney transplant (59 %, 48 %, 35 %). **B. Kaplan-Meier Survival Curve for overall survival within 2000 days**. Patients were grouped by timing of kidney transplant in relation to timing of intestinal transplant. Number at risk displayed by group in 500-day intervals. Censored data marked with a cross. Log-rank analysis of curve revealed significant differences between groups (p = 0.033). 1-, 3-, and 5-year survival ratios by group were highest for delayed kidney group (100 %, 100 %, 100 %), lower for no kidney (63 %, 63 %, 63 %), and lowest for simultaneous kidney transplant (60 %, 52 %, 37 %).

For patients with concomitant renal failure on dialysis, delayed kidney transplantation produced both better intestinal graft and patient survival compared to patients who received simultaneous kidney grafts or no kidney transplantation at all. Kaplan-Meier survival curves were constructed to estimate graft failure-free survival and overall survival (Fig. 1). DK patients had greater overall survival (p = 0.029). 1-, 3-, and 5-year survival ratios by group were highest for delayed kidney group (100 %, 100 %, 100 %, respectively), lower for no kidney (63 %, 63 %, 63 %), and lowest for simultaneous kidney transplant (60 %, 52 %, 37 %).

On log-rank analysis, DK patients also had the highest graft failure-free survival (p = 0.020). 1-, 3-, and 5-year graft failure-free survival ratios by group were highest for delayed kidney group (100 %, 100 %, 100 % respectively), lower for no kidney (63 %, 63 %, 50 %), and lowest for simultaneous kidney transplant (59 %, 49 %, 35 %).

**Fig. 2:**
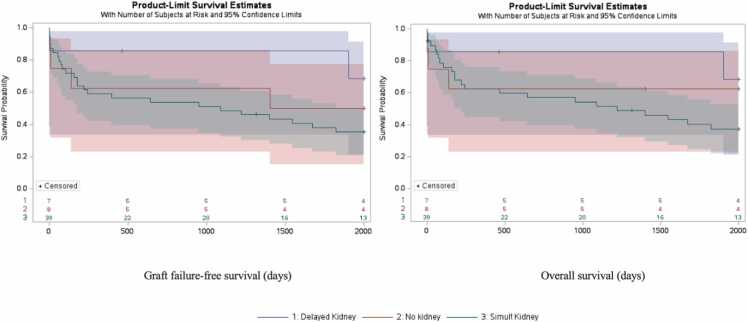
**Kaplan-Meier Survival Curve for graft failure-free survival (A) and overall survival (B) after KTx within 2000 days**. Patients were grouped by timing of kidney transplant in relation to timing of intestinal transplant. Number at risk displayed by group in 500-day intervals. Censored data marked with a cross. Log-rank analysis of curve revealed no significant differences between groups (p = 0.300, p = 0.312), although delayed kidney recipients had slightly longer survival.

We also accounted for survival bias in the delayed kidney group, since this group by definition had 100 % survival for a prolonged period of time until they received their delayed kidney transplant. Two subanalyses were carried out to identify indications of survival bias. Fig. 2 illustrates graft (Fig. 2a) and overall survival curves (Fig. 2b) where survival time for delayed kidney was adjusted to start at time of the delayed kidney transplant. Although statistical significance for both graft and overall survival was lost after this adjustment, delayed kidney recipients still showed a clinically significant trend towards longer survival.

**Fig. 3:**
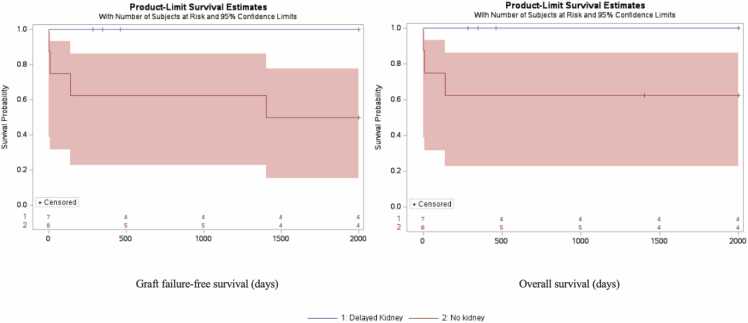
**Kaplan-Meier Survival Curve for graft failure-free survival (A) and overall survival (B) after ITx transplantation for NK and DK patients censored at time of KTx.** Patients were grouped by timing of kidney transplant in relation to timing of intestinal transplant. Number at risk displayed by group in 500-day intervals. Censored data marked with a cross. Log-rank analysis of curve revealed that NK patients were not different from DK patient in graft failure and mortality, but trended towards significance (p = 0.051, p = 0.082).

A second subgroup analysis evaluating for survival bias was performed such that graft and overall survival of DK was compared to NK (Figs. 3a, 3b). DK patients were censored at time of receiving delayed kidney grafts. The aim of this subanalysis was to identify whether there were differences in survival between NK patients and DK patients prior to receiving their kidney transplants. The survival curves for graft failure free (p = 0.051) and overall survival (0.082) trended towards significance. This trend may have been driven by 3 early deaths in the NK group before the 1-year time point. Following the early deaths, the survival curves of NK and DK in both graft and overall survival were similar.

## Discussion

There is no existing consensus, or even prior data analysis, regarding how to address the presence of pre-operative renal failure in patients listed for intestinal transplantation. Therefore, while the timing of co-transplantation of the kidney with other solid organs has been better studied, especially in combination with the liver, in which case simultaneous liver-kidney transplants have been shown to be associated with improved post-transplant survival [13], there is no literature to guide clinical decision making about the ideal timing of kidney transplantation with intestinal transplantation [14]. We addressed this gap in knowledge and identified several key results from patients undergoing both kidney and intestine transplants.

Our results show that, for intestinal transplant patients with concomitant renal failure, delayed kidney transplantation improves overall survival and graft failure-free survival compared to both simultaneous kidney-intestine transplant and intestine transplant alone at 1-, 3-, and 5-year time points. When adjusted for the effects of age and BMI, delayed kidney transplant still shows a significantly decreased hazard ratio for overall survival and graft failure-free survival compared to intestine transplant alone.

While we cannot assess specific clinical factors that influence these outcomes based on the SRTR data, we hypothesize that kidney transplants imparted more meaningful clinical benefit when performed at a time when the recipient is healthier and more hemodynamically stable than at the time of intestinal transplantation, when the kidney graft may be negatively affected by hemodynamic instability, vasopressor requirement, high calcineurin inhibitor levels, large fluid balance shifts, and frequent sepsis episodes.

Our study has several limitations. One is the small size of our study population. However, we note that it is not possible to increase the sample set since we are already using all available SRTR data. We also note that our study includes more adults than children, which might bias the findings. Given the small populations, we could not perform subgroup analysis. We also note limitations around the data on dialysis and diabetes. In addition to missing data, which limited our sample pool, there was also no available information on duration or consistency of diabetes and dialysis, which are known to impact outcomes. For patients with especially delayed kidney transplants, the data does not provide context on reasoning behind this choice. We also cannot confirm that all patients on dialysis at the time of intestinal transplantation had chronic renal failure rather than acute renal failure, however we accounted for this by including only patients who were listed for kidney transplantation at the time of intestinal transplantation, which strongly implies that all were considered to have end stage renal disease. Additionally, we did not account for an effect that having a different donor for a delayed kidney transplant might have as a secondary sensitizing event. Finally, our data set does not reveal the clinical decision making as to whether to perform simultaneous kidney transplantation versus deferring the kidney transplant.

## Conclusion

Despite these limitations, our study still shows strong support for delaying kidney transplantation for patients with end stage renal disease who are undergoing intestinal transplantation. Our findings, using the largest available data set, suggest that delayed kidney transplant confers the greatest improvement to long term overall and graft failure-free survival when compared to simultaneous kidney-intestine transplant or intestine transplant without kidney transplantation. While these results may be enough to influence clinical decision making for this patient population, they should not override the clinical judgement of the patient care team based on unique characteristics of each patient or the capabilities and resources of the center/program, and we believe that our conclusions should be validated by further study as more data becomes available.

## Ethical Clearance

Due to the use of deidentified retrospective, nationwide data provided by the Scientific Registry of Transplant Recipients (SRTR), ethical clearance was not required. Request to utilize this retrospective data for research was reviewed and approved by SRTR.

## Funding

This research did not receive any specific grant from funding agencies in the public, commercial, or not-for-profit sectors.

## CRediT authorship contribution statement

**Youjia Li:** Methodology, Investigation, Formal analysis. **Keli Wang:** Methodology, Investigation, Formal analysis. **Shaheed Merani:** Writing – review & editing. **Josh Weiner:** Writing – review & editing, Supervision, Methodology, Conceptualization. **Simran Shah:** Writing – original draft, Investigation, Formal analysis, Data curation. **Julie Hong:** Writing – original draft, Methodology, Investigation, Formal analysis.

## Declaration of Competing Interest

The authors declare that they have no known competing financial interests or personal relationships that could have appeared to influence the work reported in this paper.
